# First FHM3 mouse model shows spontaneous cortical spreading depolarizations

**DOI:** 10.1002/acn3.50971

**Published:** 2019-12-27

**Authors:** Nico A. Jansen, Anisa Dehghani, Margot M. L. Linssen, Cor Breukel, Else A. Tolner, Arn M. J. M. van den Maagdenberg

**Affiliations:** ^1^ Department of Human Genetics Leiden University Medical Center Leiden The Netherlands; ^2^ Department of Neurology Leiden University Medical Center Leiden The Netherlands

## Abstract

Here we show, for the first time, spontaneous cortical spreading depolarization (CSD) events – the electrophysiological correlate of the migraine aura – in animals by using the first generated familial hemiplegic migraine type 3 (FHM3) transgenic mouse model. The mutant mice express L263V‐mutated *α*1 subunits in voltage‐gated Na_V_1.1 sodium channels (*Scn1a* ^L263V^). CSDs consistently propagated from visual to motor cortex, recapitulating what has been shown in patients with migraine with aura. This model may be valuable for the preclinical study of migraine with aura and other diseases in which spreading depolarization is a prominent feature.

## Introduction

Cortical spreading depolarization (CSD) has been implicated in various human diseases, not only in migraine with aura and its monogenic subtype familial hemiplegic migraine, but also in stroke and traumatic brain injury.^1^ Most of the evidence linking CSD with migraine comes from animal studies, but until now CSD events, without exemption, need to be evoked by stimulating the cortex of the animal. The lack of evidence for spontaneous events in animals hampers the study of CSD initiation.

FHM3, an autosomal dominant migraine with aura subtype with severe aura symptoms, is caused by specific missense mutations in the *SCN1A* gene that encodes the *α*1 subunit of voltage‐gated Na_V_1.1 sodium channels.^2^ The overwhelming majority of mutations in *SCN1A* cause Dravet syndrome, a childhood epilepsy phenotype, due to loss of channel function.^3^ FHM3 mutations instead seem associated with gain of Na_V_1.1 channel properties, as was demonstrated in heterologous expression studies.^4^, ^5^, ^6^ The FHM3 L263V mutation resulted in impaired channel inactivation, accelerated recovery from inactivation, and greater channel availability of the mutant channels in tsA‐201 cells.^6^

To study in vivo effects of the L263V mutation, and assess whether mutant mice exhibit spontaneous CSD, we generated the first FHM3 knock‐in mouse model by introducing the mutation into the endogenous *Scn1a* gene using a CRISPR/Cas9 approach. We present the *Scn1a* ^L263V^ mouse model as a promising mouse model to further the understanding of disease mechanisms in which spreading depolarizations are involved.

## Methods

### Generation of *Scn1a* ^L263V^ mice

A 1.3 kb dsDNA fragment containing a C to G transition at nucleotide position 787 in exon 7, resulting in a leucine to valine (L263V) amino acid change, of the *Scn1a* gene was used as a template for CRISPR/Cas9‐mediated homologous recombination, and introduced in JM8 (C57BL/6J) embryonic stem cells. Clones carrying the correct mutation were injected in C57BL/6J blastocysts to generate chimeric mice. The L263V mutation was transmitted through the germline by breeding chimeric mice with C57BL/6J mice and the subsequent line was maintained on the same genetic background. As maintaining other *Scn1a* mutants on a 129/SvJ background increased survival,^7^ we also interbred our mutants with 129/SvJ mice. On either background success of breeding was greatly enhanced by feeding male heterozygous *Scn1a* ^L263V^ mice chow that contained GS967 (8 mg/kg chow; Research Diets, New Brunswick, NJ), as previously used.^8^, ^9^ During the mating period, males were given standard chow, just as female wild‐type (WT) mice. Hence all experimental mice were exclusively exposed to standard chow. Experiments were approved by local and national ethical committees conforming to the recommendations of the European Communities Council Directive (2010/63/EU) and carried out in accordance with ARRIVE guidelines. For behavioral phenotyping, naïve *Scn1a* ^L263V^ and WT mice were videotaped from P21–P28 for 2 months or until death.

### Surgery for electrophysiology

Prior to surgery, all surgical instruments and electrodes were disinfected with 70% ethanol. ECoG (silver balltips; 75 *µ*m, AG5493; Advent Research Materials, Oxford, UK) or intracortical electrodes (75 *µ*m platinum/iridium, PT6718) were implanted in *Scn1a* ^L263V^ (*n* = 15 and *n* = 9, respectively) and WT (*n* = 8 and *n* = 5, respectively) mice (P21–P28) under isoflurane anesthesia (4% for induction; 1.5% for maintenance) bilaterally in the primary visual cortex (V1; −3.5/2.0/0.5; mm relative to bregma; anterior, lateral, and ventral, respectively) and primary motor cortex (M1; +1.5/1.8/0.5). In a subset of animals (14/24 *Scn1a* ^L263V^ and 9/13 WT mice), an additional electrode was implanted in the right hippocampus (−2.2/1.5/1.7).

In a further 8 *Scn1a* ^L263V^ mice, epicranial electrodes were placed by thinning of the skull overlying V1 and M1, followed by attachment of silver ball tips using conductive carbon‐based glue (Anders Products, Melrose, MA).

Electrical threshold for CSD was established by cortical stimulation in *Scn1a* ^L263V^ (*n* = 9) and WT (*n* = 10) mice. Bipolar stimulation electrodes were implanted bilaterally in caudal V1 (−3.8/2.3/0.5), with recording electrodes in rostral V1 (−2.5/2.4/0.5), M1, and the right hippocampus.

### Data acquisition and analyses

Following surgery, animals were connected to a custom‐made 7‐channel system for continuous video‐EEG recordings. Data were acquired as described previously.^10^ CSD was defined as a transient negative DC‐shift of >5 mV measured at two locations with a delay, associated with a decrease in AC amplitude. Cortical DC‐shifts in only one electrode accompanied by decreased AC amplitude were never observed. DC‐recordings were inspected for CSDs during 2 weeks postoperatively, or until death of the animal. For all *Scn1a* ^L263V^ mice, abnormal behavior, such as seizures, were studied for the whole recording period using AC‐recordings and video.

For CSD induction, cathodal pulses of increasing intensities (1–5000 *µ*C) were delivered every 3 min, until a CSD was observed, and propagation speed between electrodes in V1 and M1 was calculated. Two stimulations were performed per animal, at 24 and 48 h after surgery (once in either hemisphere), and threshold and propagation rate were averaged for analyses.

Statistical testing was performed in Graphpad Prism (GraphPad Software, La Jolla, CA). A *P*‐value of <0.05 was considered significant.

### Immunohistochemistry

Mice were perfused with PBS and 4% PFA. Brains were postfixed, cryoprotected, and coronally sectioned (20 *µ*m) on a cryostat. Antigen retrieval in 10 mmol/L citrate buffer with 0.05% Tween was followed by blocking in 10% normal goat serum for 90 min and double‐labeling with rabbit anti‐Na_v_1.1 (1:200; Alomone Labs, Jerusalem, Israel) and mouse anti‐GAD67 (1:200; Millipore Sigma, St. Louis, MO) antibodies overnight at 4°C. Incubation in goat anti‐rabbit Cy2 (1:200) and goat anti‐mouse Cy3 (1:200; Jackson Immunoresearch, Cambridgeshire, UK) antibodies was performed for 2 h. Sections were mounted in medium containing 1 *µ*L/mL Hoechst‐33258 and examined using confocal microscopy.

## Results

### Generation of *Scn1a* ^L263V^ mice

**Figure 1:**
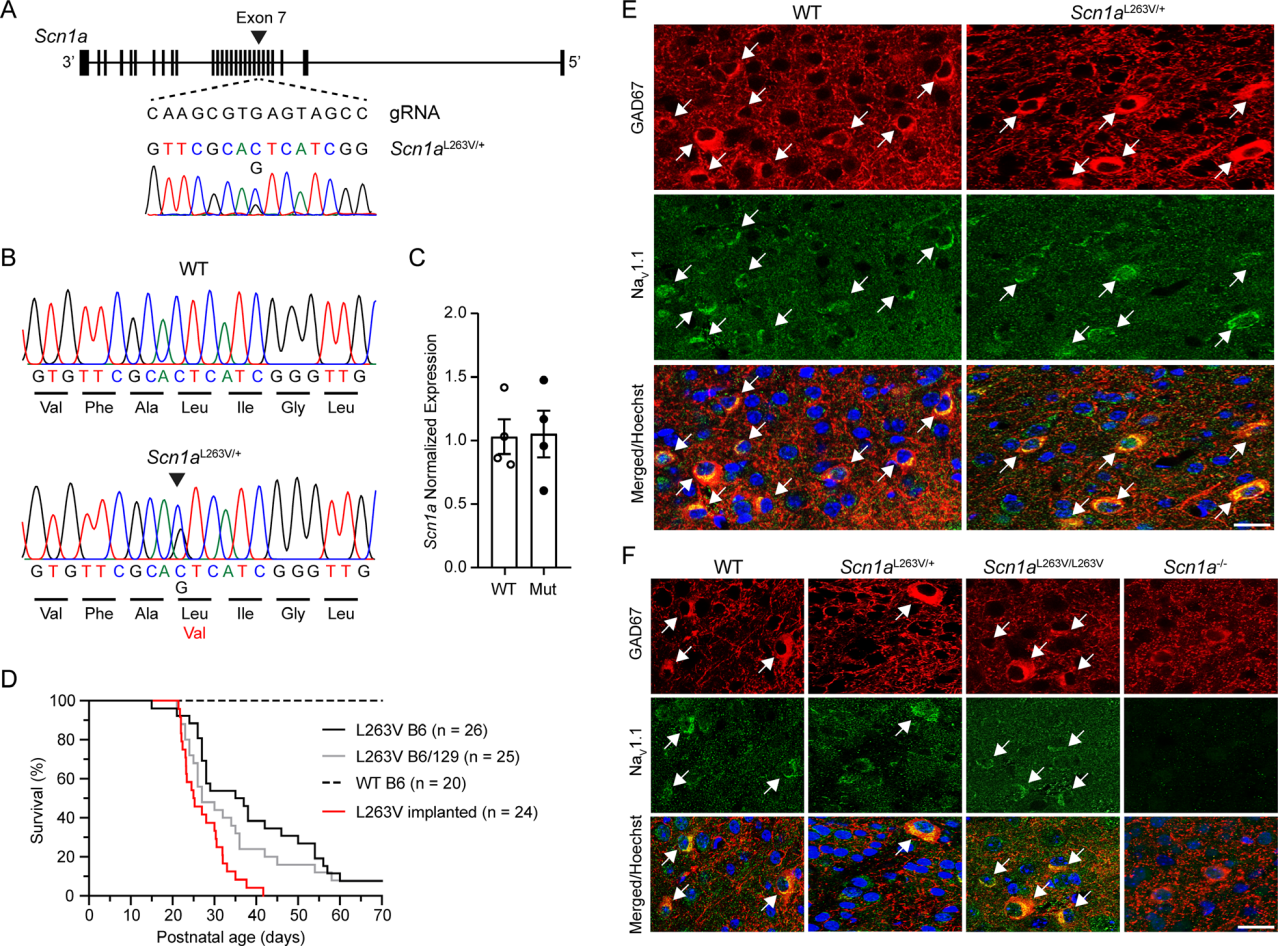
Generation, molecular characterization, and survival of *Scn1a* ^L263V^ mice. (A) Genomic structure of the *Scn1a* allele and guide RNA used to introduce the L263V mutation in exon 7 (indicated by the arrowhead) and the electropherogram of the modified DNA sequence. (B) Sequencing analysis of RT‐PCR products from whole brain mRNA isolated from WT and heterozygous *Scn1a* ^L263V^ mice. (C) Normalized expression of *Scn1a* mRNA in WT and *Scn1a* ^L263V^ (Mut) mice. (D) Survival of naïve (not implanted) *Scn1a* ^L263V^ mice on a C57BL/6J (B6; black) and mixed (50:50) C57BL/6J/129/SvJ (B6/129; grey) background was not significantly different (*P* = 0.152, Log‐rank test). Implanted C57BL/6J *Scn1a* ^L263V^ mice (red) had a significantly decreased survival (*P* < 0.001, Log‐rank test), whereas no early mortality was observed in implanted WT mice (*n* = 9; not shown). (E,F) Immunofluorescence of Na_V_1.1 protein in the primary visual cortex of P21 WT and heterozygous *Scn1a* ^L263V^ (E) and P14 WT, heterozygous and homozygous *Scn1a* ^L263V^ (F) mice showed a strong overlap in expression with GAD67, a marker for inhibitory interneurons (white arrows indicating double‐labeled neurons) that was similar between genotypes. Scale bars, 20 *µ*m.

CRISPR/Cas9‐mediated homologous recombination was used to introduce the human FHM3 L263V missense *SCN1A* mutation in the orthologous mouse gene, generating *Scn1a* ^L263V^ mice (Fig. 1A). Cortical expression of Na_V_1.1 protein was observed, mostly in GABAergic interneurons, in WT and heterozygous *Scn1a* ^L263V^ mice at P21 (Fig. 1E), and in WT, heterozygous, and homozygous *Scn1a* ^L263V^ mice at P14 (Fig. 1F) with no overt difference between genotypes.

### Survival of *Scn1a* ^L263V^ mice

Heterozygous *Scn1a* ^L263V^ mice died at juvenile to young adult age, irrespective of the genetic background (C57BL/6J or mixed (50:50) C57BL/6J/129/SvJ) (Fig. 1D). Video recordings of both naïve (*n* = 11; 8 females) and implanted (*n* = 24; 17 females) animals revealed that fatality was preceded by limited abnormal behavior lasting 1–14 sec characterized by hindlimb jerks and/or tonic hindlimb extension sometimes preceded by sudden wild running, or occurred directly from sleep (*n* = 3). No seizure‐related or other abnormal behaviors were observed over the recording period preceding death.

### Spontaneous and induced CSD in *Scn1a* ^L263V^ mice

**Figure 2:**
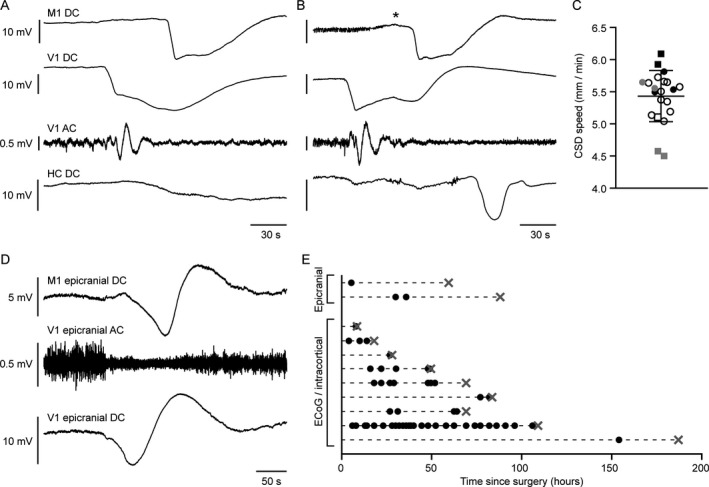
Spontaneous cortical spreading depolarization (CSD) in *Scn1a* ^L263V^ mice with propagation from visual to motor cortex. (A and B) Examples of a spontaneous CSD during wakefulness in an *Scn1a* ^L263V^ mouse, without evidence of spread to the ipsilateral hippocampus (HC) (A) and during sleep, with spread to the ipsilateral HC (B) (M1 = primary motor cortex; V1 = primary visual cortex). In the example in B, the animal awoke during the CSD (indicated by an asterisk), but no overt abnormal behavior was observed. (C) Propagation rate of spontaneous CSDs occurring >24 h following surgery. Every symbol represents a single CSD, grouped per animal (squares represent males, total *n* = 21 CSDs in 6 mice). (D) Example of a spontaneous CSD detected by epicranial electrodes in an *Scn1a* ^L263V^ mouse. (E) Incidence of spontaneous CSDs recorded with ECoG, intracortical or epicranial electrodes. Each row represents one mouse, with individual CSDs indicated by black dots and death indicated by a gray cross. With respect to the time since surgery, CSD incidence was similar between the first and second half of recordings (26 and 27 CSDs, respectively). Note that epicranial recordings were not included in the analysis of CSD propagation speed (C), as onset of the DC‐shift appeared more gradual. For time series of V1 AC recordings (A,B,D) a bandpass filter of 0.5–100 Hz was used.

In a subset of *Scn1a* ^L263V^ mice (9/24; 6 females; ECoG in 5/9, intracortical in 4/9), a total of 50 spontaneous CSD events were observed in 601 h of recording (compared to 0/13 WT mice; *χ* ^2^(1) = 6.442, *P* = 0.011). The survival time of mice with spontaneous CSDs was not different from mice without CSDs (67 ± 24 h versus 108 ± 35 h, respectively; *P* = 0.327, unpaired *t*‐test). In animals with more than one CSD (*n* = 6; range 2–33 CSDs), time between consecutive CSDs ranged from 28 min–31.2 h. CSD events generally occurred in isolation; only one CSD event consisted of 2 DC‐shifts in each cortical electrode separated by approximately 3 min. Notably, all CSDs spread from V1 to M1 (Fig. 2A and B). In 6/9 mice with spontaneous CSDs, hippocampal DC‐potential was measured. Spread of CSDs to the ipsilateral hippocampus was observed in only one animal (Fig. 2B). In 3/9 animals with a hippocampal electrode, downward spikes of moderate amplitude (0.5–1.5 mV) were observed most often in V1, sometimes in M1, but never in hippocampus. These spikes occurred every few seconds, had a duration of 20–50 msec, and were not associated with behavioral abnormalities. M1, V1, and hippocampal recordings did not reveal seizure bursts during the fatal event or preceding recording period (total recording time of 2228 h, *n* = 24; 17 females). No overt CSD‐related behavioral abnormalities, including previously reported wet dog shakes or freezing behavior,^11^ were observed. A subset of CSDs occurred during sleep (21/50) and were accompanied by awakening of the animal (9/21), roughly coinciding with spread to M1 (example in Fig. 2B).

Although absence of spontaneous CSD in WT mice suggests that this phenomenon is specific to *Scn1a* ^L263V^ mice, we additionally tested whether spontaneous CSDs occurred in the absence of cortical damage induced by implantation of intracortical and/or ECoG electrodes. To this end, we measured DC‐potential in *Scn1a* ^L263V^ mice using epicranial electrodes that did not penetrate the skull. In 2/8 chronically recorded *Scn1a* ^L263V^ mice, 3 CSDs were detected in 146 h of recording (Fig. 2D and E).

**Figure 3:**
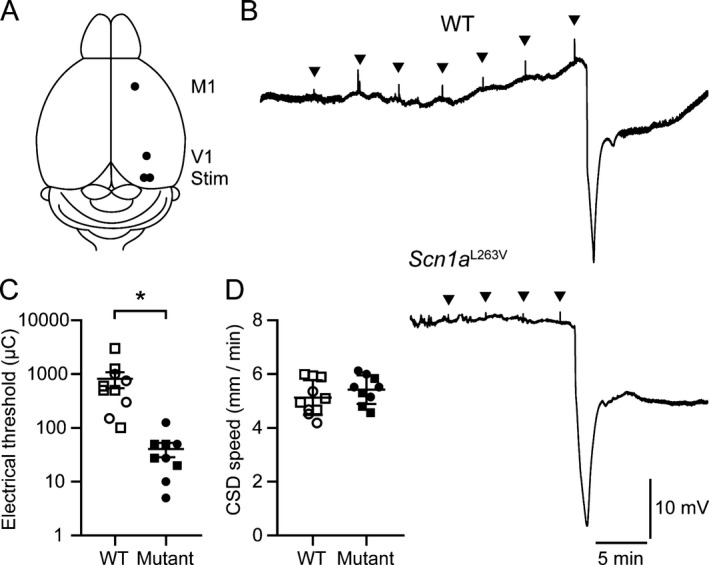
Reduced threshold for cortical spreading depolarization (CSD) induced by electrical stimulation in freely behaving *Scn1a* ^L263V^ mice. (A) Top view of experimental approach for electrical stimulation of the visual cortex (Stim; M1 = primary motor cortex; V1 = primary visual cortex). (B) Example of DC‐signal in primary motor cortex of a WT and *Scn1a* ^L263V^ mouse, showing that more stimulations of increased intensity (arrowheads) were required to induce CSD in WT mice. Group analyses showed a reduced electrical threshold for CSD in *Scn1a* ^L263V^ mice (*P < 0.001, Mann–Whitney test), while CSD propagation rate was not different (*P* = 0.29, Welch’s *t*‐test). Squares represent males.

Cortical cathodal stimulation of V1 was performed in a separate group of freely behaving mice (*n* = 9 *Scn1a* ^L263V^, 5 females; *n* = 10 WT, 4 females). CSD threshold was significantly lower in *Scn1a* ^L263V^ mice than in WT littermates, whereas no difference was observed for propagation rate (Fig. 3).

## Discussion

Investigation into the mechanisms involved in initiation of CSD events is hampered by the fact that such events need to be evoked as there is no animal model, yet, that shows spontaneous occurrence of CSD in metabolically intact tissue. Here, we report that mice expressing the human *SCN1A* ^L263V^ mutation, that was shown to cause FHM3 in humans, exhibit spontaneous CSDs that propagate from visual to motor cortex.

Functional consequences of FHM3‐related *SCN1A* mutations in transfected cells indicate an overall gain of function of Na_V_1.1 channels.^4^, ^5^, ^6^ This sharply contrasts with the loss of function observed with mutations causing Dravet syndrome, resulting in loss of Na_V_1.1 expression predominantly affecting GABAergic interneurons.^7^, ^12^, ^13^, ^14^ In mice, this loss of function results in spontaneous seizures and large‐amplitude interictal spikes, which we did not observe in *Scn1a* ^L263V^ mice. As Na_V_1.1 is predominantly localized in inhibitory interneurons,^7^, ^14^ the present data suggest that the prominent CSD phenotype in *Scn1a* ^L263V^ mice results from hyperexcitable cortical inhibitory interneurons. This mechanism seems counterintuitive in light of reports that GABA‐A receptor activation could inhibit, not facilitate, CSD.^15^, ^16^, ^17^ Still, a recent *in silico* study indeed suggested that intense firing of inhibitory interneurons may induce CSD, because of accumulation of extracellular potassium.^18^ Of note, increased sodium currents have also been reported in excitatory neurons of loss‐of‐function *Scn1a* mutants^19^ and increased persistent sodium currents, which may contribute to CSD^20^ have been reported in transfected cells that expressed *SCN1A* mutations associated with Dravet syndrome^21^ and FHM3.^6^ Clearly, the cellular substrate underlying CSD susceptibility in *Scn1a* ^L263V^ mice remains to be determined.

Unexpectedly, all *Scn1a* ^L263V^ mice died prematurely with peak mortality between P21–35. Contrary to previous studies on loss‐of‐function *Scn1a* mutants,^7^, ^22^ genetic background, that is, maintaining the mutation on either a pure C57BL/6J or the mixed (50:50) C57BL/6J/129/SvJ background, had no significant impact on survival of *Scn1a* ^L263V^ mice. Timing of peak mortality, however, seems to overlap with that of loss‐of‐function *Scn1a* mutants.^23^ This may be related to a developmental peak in Na_V_1.1 expression in this age range.^14^ In addition, spreading depolarization susceptibility is particularly high in this developmental time window^24^, ^25^ and was found to induce lethal apnea in an FHM1 mouse model.^10^, ^26^ However, as death is seizure‐related in FHM1 mutants^10^, ^26^ and loss‐of‐function *Scn1a* mutants,^23^ the mechanism of death in *Scn1a* ^L263V^ mice may be different.

Notably, in *Scn1a* ^L263V^ mice, all spontaneous CSDs spread from visual to motor cortex, which is in line with rare observations of visual aura features from neuroimaging in patients with migraine with aura,^27^ commonly attributed to the occurrence of CSD. Together, these data indicate that *Scn1a* ^L263V^ mice may serve as a valuable model to study mechanisms underlying initiation of spreading depolarizations, which may be relevant to disorders including migraine with aura, stroke and traumatic brain injury.

## Author Contributions

N.A.J. conceptualized the study, designed and performed experiments, analyzed the data, wrote, and revised the manuscript. A.D. performed histology and confocal microscopy. M.M.L.L. maintained the mouse colony and performed molecular analyses. C.B. performed CRISPR/Cas9‐mediated mutagenesis and molecular analyses. E.A.T. advised on experiments and revised the manuscript. A.M.J.M.v.d.M. conceptualized the study, advised on experiments and revised the manuscript.

## Conflict of Interest

Nothing to report.
